# Safety and efficacy of drug-eluting bead transarterial chemoembolization with CalliSpheres® microsphere for hepatocellular carcinoma with portal vein tumor thrombus: a preliminary study

**DOI:** 10.7150/jca.54650

**Published:** 2021-06-01

**Authors:** Tan-Yang Zhou, Sheng-Qun Chen, Hong-Liang Wang, Sheng-Ming Weng, Guan-Hui Zhou, Yue-Lin Zhang, Chun-Hui Nie, Tong-Yin Zhu, Bao-Quan Wang, Zi-Niu Yu, Li Jing, Feng Chen, Jun-Hui Sun

**Affiliations:** 1 Hepatobiliary and Pancreatic Interventional Treatment Center, Division of Hepatobiliary and Pancreatic Surgery, The First Affiliated Hospital, Zhejiang University School of Medicine, Hangzhou, Zhejiang Province, China.; 2 Zhejiang Provincial Research Center for Diagnosis and Treatment of Hepatobiliary Diseases, Hangzhou, Zhejiang Province, China.; 3 Zhejiang Clinical Research Center of Hepatobiliary and Pancreatic Diseases, Hangzhou 310003, Zhejiang Province, China.; 4 Department of Radiology, Jingning County People's Hospital, Lishui, Zhejiang Province, China; 5 Department of Radiology, The First Affiliated Hospital, Zhejiang University School of Medicine, Hangzhou, China.

**Keywords:** Hepatocellular carcinoma, Chemoembolization, Therapeutic, Microspheres, Prognosis

## Abstract

**Objective**: To prospectively evaluate the safety and therapeutic effectiveness of drug-eluting beads transcatheter arterial chemoembolization (DEB-TACE) with CalliSpheres® microsphere (CSM) for the treatment of hepatocellular carcinoma (HCC) with portal vein tumor thrombus (PVTT), and to analyze the prognostic factors.

**Method**: Between November 2015 and November 2017, consecutive 58 HCC patients with PVTT who received DEB-TACE with CSM treatment were prospectively enrolled in this study. The demographic characteristics, adverse events (AEs) and treatment response were collected. Overall survival (OS) and progression-free survival (PFS) were calculated using the Kaplan-Meier method. Univariate and multivariate Cox regression analyses were performed to determine the independent factors correlated with OS.

**Results**: The objective response rate (ORR) was 79.3% in terms of tumors and 44.8% in thrombi. The median PFS and OS of patients were 5.0 months and 9.0 months respectively. The cumulative survival rate at 3-, 6-, 9-, 12-, 18- and 24-month were 94.8%, 72.4%, 53.4%, 41.4%, 22.4% and 19.0%, respectively. In a stepwise multivariate Cox proportional hazards model, the higher Child-Pugh classification (HR=2.279; 95%CI, 1.042-4.985, p = 0.039) and tumor burden (p = 0.008) were the significant predictors of poorer OS after adjustment for known risk factors. The most common clinical AEs were postembolization syndrome (PES) and the most prevalent laboratory toxicity was transient liver function damage.

**Conclusion**: DEB-TACE with CSM is safe and well-tolerated in HCC patients with PVTT, and reveals a favorable preliminary clinical outcome. The higher Child-Pugh classification and liver tumor burden are independent prognostic factors associated with poor survival for HCC patients with PVTT treated by DEB-TACE with CSM.

## Introduction

Hepatocellular carcinoma (HCC) is the most common tumor in the hepatobiliary system and is the third leading cause of cancer death all around the world 1. HCC tends to invade the intrahepatic vasculature, especially the portal vein and form a portal vein tumor thrombus (PVTT)^2^. PVTT is associated with a dismal prognosis for HCC patients, with a median survival time of only 2.7 months without any interventions 3.

Current therapeutic approaches for HCC patients with PVTT include hepatectomy, transcatheter arterial chemoembolization (TACE), radiotherapy, systematic chemotherapy according to the national guidelines in China 4. Among these available treatment approaches, TACE is one of the most commonly used techniques to manage unresectable HCC with PVTT 5. However, most of the previous studies mainly using conventional TACE (cTACE), which the rationale behind is that the intra-arterial chemotherapy using lipiodol and chemotherapeutic agents, followed by selective vascular embolization, will result in a strong cytotoxic effect combined with ischemic necrosis. Recently, drug-eluting beads transcatheter arterial chemoembolization (DEB-TACE) was developed to treat advanced HCC and was known to deliver higher doses of chemotherapeutic agents and to prolong contact time with the tumor 6. However, there are limited data are available concerning the use of DEB-TACE in HCC patients with PVTT.

CalliSpheres® microsphere (CSM), is a newly invented drug-eluting beads (DEB) in China, which is structured on a polyvinyl alcohol hydrogel modified with sulfonate groups. Many pieces of research have proved that it can load more kinds of chemotherapy drugs and has a higher drug-loading efficiency compared with other DEBs 7-9. According to several recent studies, unresectable HCC patients who receive DEB-TACE treatment using CSM have better treatment response, prolonged overall survival (OS) and reduced adverse events (AEs) compared with cTACE^10^-^11^. Therefore, the purpose of this study was to investigate the treatment efficacy and safety of DEB-TACE using CSM and to identify important prognostic factors related to the survival of HCC patients with PVTT.

## Methods

### Patients

Between November 2015 and November 2017, consecutive 58 HCC patients with PVTT who received DEB-TACE treatment using CSM in the First Affiliated Hospital, Zhejiang University School of Medicine were prospectively enrolled in this study. The inclusion criteria were as follows: (1) diagnosed as HCC confirmed either by clinical and histopathological examinations according to the American Association for the Study of Liver Diseases (AASLD) 12; (2) PVTT was confirmed by enhanced Computed Tomography (CT) or enhanced Magnetic Resonance Imaging (MRI) according to Chinese Expert Consensus on Multidisciplinary Diagnosis and Treatment of Hepatocellular Carcinoma with Portal Vein Tumor Thrombus (2018 Edition) 5 and the imaging features of PVTT included solid lesions within the portal vein in all the phases of intravenous enhanced 3-phase computed tomography, especially with an enhancement of contrast in the arterial phase and washout in the portal venous phase of the procedure. PVTT was divided according to Cheng's PVTT classification system into the following categories 5: type I, tumor thrombus involve the segmental branches of the portal vein or above; type II, the right/left portal vein; type III, the main portal vein; and type IV, the superior mesenteric vein; (3) with Eastern Cooperative Oncology Group (ECOG) performance status ≤1 and Child-Pugh classification A or B; (4) medical records were completely reserved and available. The exclusion criteria included: (1) patients with obvious hepatic arteriovenous fistula showed in CT/MRI or decompensated cirrhosis; (2) patients who accompanied with severe cardiovascular diseases or other malignancies; (3) patients whose medical records were incomplete; (4) patients with a history of either locoregional therapies or systemic treatments before enrolled. The present study was approved by the Institutional Review Board of the First Affiliated Hospital, Zhejiang University School of Medicine, with informed consent obtained from all patients.

### Data collection

All data were extracted from the electronic medical records. Baseline demographics and clinical characteristics included age, gender, ECOG performance status, Child-Pugh classification, viral etiology, Hepatitis B Virus (HBV)-DNA, liver cirrhosis, ascites, number of HCC nodules, macroscopic classification, maximum tumor diameter, tumor burden, serum alpha-fetoprotein (AFP), PVTT classification and extrahepatic metastasis. Besides, treatment response, routine laboratory tests (including complete blood count, biochemical parameters) before and after treatment (4-6 weeks) were also collected.

The tumor burden, the percentage of total tumor in the liver, was independently determined by an abdominal radiologist with at least 3 years of experience in liver imaging on a workstation (Advantage Windows®,VolumeShare 4, GE Healthcare, Milwaukee, Wisc., USA) using dedicated software (Volume Viewer®, GE Healthcare, Milwaukee, Wisc., USA).

### DEB-TACE procedures

Before the initiation of DEB-TACE, the concentrated solution of epirubicin was prepared with 4 ml of sterile water for injection and 80 mg of epirubicin hydrochloride, and the concentration was 20 mg/ml. Then the CSM (Jiangsu Hengrui Medicine Co., Ltd., Jiangsu Province, China) with sizes of 100-300 μm were loaded with epirubicin as follows: firstly, the CSM and sterile water were extracted by a 20 mL syringe and inverted placed for 5 min until the CSM were totally precipitated, then pushed out the supernatant liquor. Subsequently, the concentrated solution of epirubicin was mixed with the CSM using a tee joint and then stored by a syringe, and the syringe containing the mixture of CSM and epirubicin solution was placed at room temperature and shaken gently every 5 min until almost all epirubicin were loaded (loading time more than 30 min). After that, the nonionic contrast medium (iodixanol [320 mg I/mL], Jiangsu Hengrui Medicine, Jiangsu, China) was added into the mixture as a 1:1 ratio and the mixture were kept still for 5 min for further application.

It was recommended to use 2.4-F to 2.8-F microcatheters for the transcatheter embolization of tumor feeders, to perform cone-beam CT as soon as deemed necessary, and to inject the beads slowly (ideally 1 mL/min) through a 1-mL syringe until the reduced flow of the feeding artery with the conventional method of two to five heartbeats to clear the contrast column from the microcatheter tip. Additional embolic material such as polyvinyl alcohol (PVA) particles or tris-acryl gelatin microspheres (Embosphere® Microspheres, BioSphere Medical, Rockland, MA) was used if the beads were completely delivered before the endpoint of embolization was reached. Superselective embolization as much as possible was strongly recommended to avoid injury of nontumoral liver parenchyma.

All patients received routine tests including complete blood count, biochemical indexes, coagulation function, tumor markers and liver enhanced CT or MRI before and after DEB-TACE. Besides, the necessary symptomatic and supportive treatments such as actively liver-protecting treatment, pain relief, antiemetics and prophylactic anti-infection were provided to all patients after DEB-TACE until they fully recovered and discharged.

### Safety assessment

The safety assessment was evaluated based on a procedure related to AEs grading. AEs were assessed within 1 month after the procedure and included clinical and laboratory toxicity. The grading of AEs were defined as low (grades 1-2) or high (grades 3-4) according to Common Terminology Criteria for Adverse Events v5.0 (CTCAE v5.0).

### Response assessment and follow up

Liver enhanced CT or MRI examination was performed at 4-6 weeks after DEB-TACE, and the treatment response was assessed according to modified Response Evaluation Criteria in Solid Tumors (mRECIST), which included complete response (CR), partial response (PR), stable disease (SD) and progressive disease (PD). The PVTT radiologic response was assessed as a complete response (CR, without any enhancement or complete disappearance), partial response (PR, >50% decrease in the enhancement region or the thrombus diameter), stable disease (SD, <50% decrease or<25% increase in the enhancement region or the thrombus diameter or cavernous transformation), and progressive disease (PD, >25% increase in the thrombus diameter or newly developed PVTT), based on the criteria described by Yoon et al 13. Overall response rate (ORR) was defined as the rate of CR+PR. Moreover, patients were followed up by calls, outpatient service and hospitalizations for 6-30 months after DEB-TACE treatment, and the last follow-up date was Jun 30^th^, 2020. PFS was defined as the time elapsed between DEB-TACE treatment initiation and tumor progression or death from any cause, with censoring of patients who received other therapies or were lost to follow-up. OS was defined as the period from the first DEB-TACE treatment to the date of death from any cause or until the date of the last follow-up.

### Statistical analysis

Count data were expressed as count (percentage), and the measurement data were presented as mean ± standard deviation. Comparison of measurement data between pretreatment and posttreatment was determined by paired t-test. OS was analyzed using the Kaplan-Meier method and compared using the log-rank test. The multivariate Cox proportional hazard modeling was performed to identify independent prognostic factors based on the adjusted hazard ratio and its associated 95% confidence interval (CI). All statistical analyses were performed using SPSS 22.0 (IBM,Chicago, IL, USA). For all tests, P < 0.05 was considered statistically significant.

## Results

### Patients' baseline characteristics

Among the 58 patients, 53 were men (91.4%) and 23 patients aged ≥60 years (39.7%). The majority of patients were ECOG 1(62.1%) in performance status and with Child-Pugh classification A liver function (81%). Approximate two-thirds of patients had multiple tumor nodules and type I/II PVTT. There were only 31% of patients with low tumor burden (≤30%) and 47 patients without extrahepatic metastasis. In 11 patients with extrahepatic metastasis, ten suffered from pulmonary metastasis and one had abdominal lymph node metastasis. The baseline characteristics of patients are presented in **Table 1**.

### Treatments

The 58 patients included in the study received 71 cycles of DEB-TACE. The median number of TACE sessions per patient was 1.2 (range, 1-3).The vast majority of the patients (82.8%) received one cycle and only 3 patients receive 3 cycles. In addition, of all the 58 patients, 34 cases only underwent DEB-TACE treatment, 11 cases received DEB-TACE combined with sorafenib, 4 cases underwent DEB-TACE combined with surgery, 3 cases received DEB-TACE combined with liver transplantation, 2 cases received DEB-TACE combined with portal vein iodine 125 particle stent, 2 cases received DEB-TACE combined with sorafenib and surgery, 1 case underwent DEB-TACE combined with ^131^I-metuximab injection, and 1 case underwent DEB-TACE combined with intraperitoneal hyperthermic perfusion chemotherapy.

### Treatment response evaluation

Treatment response of patients are evaluated according to the mRECIST, which showed that 3 (5.2%) and 43 (74.1%) patients achieved CR and PR respectively, with an ORR 79.3% regarding tumors; 2 (3.4%) and 24 (41.4%) patients achieved CR and PR respectively, with an ORR 44.8% in terms of tumor thrombus (**Table 2**).

### Survival evaluation of patients

**Figure 1:**
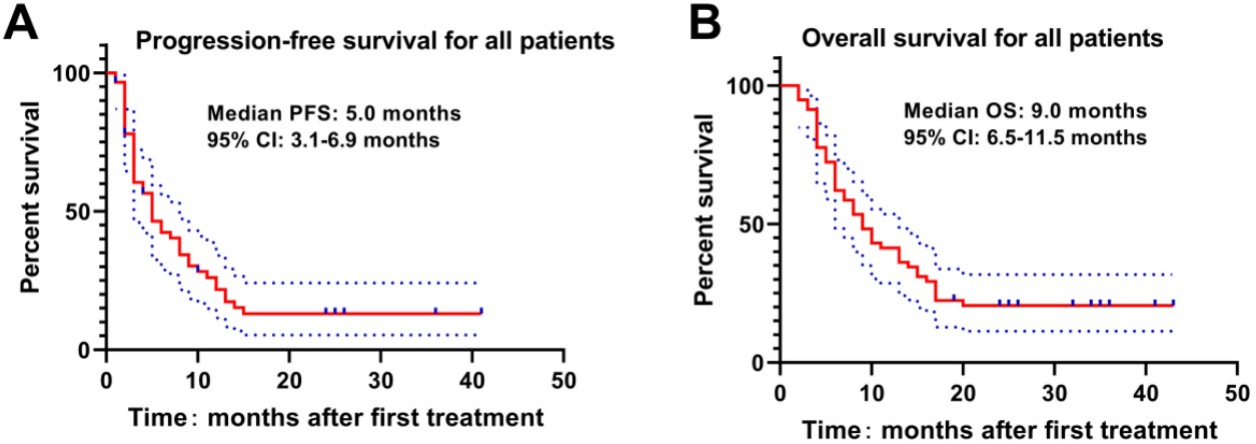
** PFS and OS of HCC patients with PVTT underwent DEB-TACE with CSM.** The median PFS(A) and OS(B) were 5.0 months and 9.0 months respectively in HCC patients with PVTT underwent DEB-TACE with CSM.

**Figure 2:**
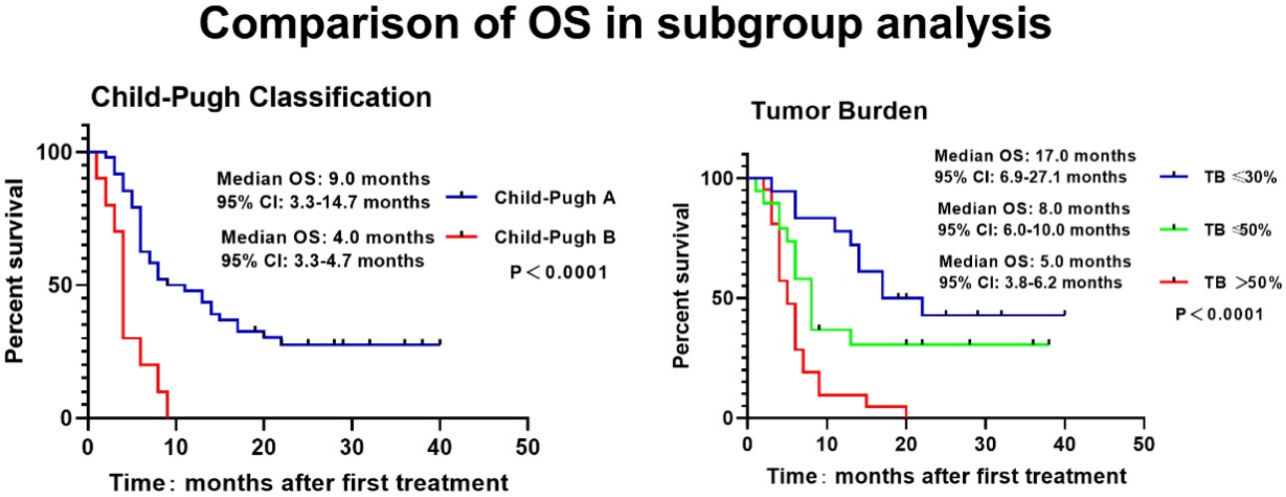
** Associations of Child-Pugh classification and tumor burden with OS.** The higher Child-Pugh classification(A) and the higher tumor burden (B) were associated with poorer OS. Survival profiles were illuminated with Kaplan-Meier method. OS, overall survival; TB, Tumor Burden

The Kaplan-Meier curve was drawn to analyze the PFS and OS of patients, which revealed that the median PFS and OS of patients were 5.0 months and 9.0 months. The 3-, 6-, 9-, 12-, 18- and 24-month OS rates were 94.8%, 72.4%, 53.4%, 41.4%, 22.4% and 19.0%, respectively (**Figure 1**). Meanwhile, higher Child-Pugh classification (**Figure 2A**) and tumor burden (**Figure 2B**) were observed to be associated with worse OS.

### Cox's proportional hazards regression model analysis of factors affecting OS

To analyze factors affecting OS, univariate Cox's proportional hazards regression model was applied, which revealed that Child-Pugh classification (P=0.004), extrahepatic metastasis (P=0.022) and larger maximum tumor size (P=0.003) , as well as tumor burden (P=0.001) were associated with poorer OS (**Table 3**). To further explore independent factors for predicting OS, multivariate Cox's proportional hazards regression model was utilized, and it showed that higher Child-Pugh classification (P=0.032) and tumor burden (P=0.002) were independent factors for predicting worse OS (**Table 3**).

### Assessment of safety

The safety assessment was performed one day after the procedure for laboratory toxicity and within one week for clinical toxicity. The detailed safety assessment is shown in Table 4. The most common AEs were moderate fever (91.4%), abdominal pain (75.9%) and nausea (70.7%), which were known as the postembolization syndrome (PES). Almost all patients had suffered transient liver function damage after the procedure, with mild to moderately elevated alanine transaminase (50.0%) and bilirubinemia (63.8%), but high-grade aspartate transaminase elevation (58.6%). No deaths occurred within 1 month after treatment. However, three patients died due to hepatic failure (*n* = 1) or gastrointestinal bleeding (*n* = 2) within 3 months after the procedure.

## Discussion

There is a high incidence of PVTT in HCC patients, which is associated with a poor prognosis 14-15. But at present, treatment strategies for HCC patients with PVTT remain controversial. According to the Barcelona Clinic for Liver Cancer (BCLC) staging system, PVTT is recognized as an advanced stage disease (BCLC-C) and systemic therapy such as sorafenib is the only recommended therapeutic strategy by the AASLD guidelines 12, but in the Asia-Pacific region TACE is an essential method for HCC patient with PVTT in many medical centers 16-17. In a recent study, the median survival time of TACE (7.49 months) was similar to the result of the SHARP trial (8.1 months) in patients with HCC and vascular invasion 17-18, which were slightly lower than that of our study. Moreover, in the SHARP trial, 95% of patients were categorized as Child-Pugh classification A liver function and tumor thrombosis was limited as macroscopic vascular invasion. Hepatic resection (HR) is a safe and effective treatment for HCC with PVTT when patients are carefully selected 19. HR can eradicate both the main tumor and satellite tumors as well as PVTT to reduce the pressure on the portal vein, preventing the occurrence of refractory ascites and bleeding of esophageal varices, protecting liver function, and reducing tumor burden as well as intrahepatic and extrahepatic metastasis of HCC 20. But type III or IV PVTT may lead to intraoperative difficulties in resecting the thrombi. Meanwhile, portal vein wall invasion may lead to thrombi residue and a high risk of postoperative recurrence. A systematic review about the safety and efficacy of HR for treating HCC involving a single large tumor (>5 cm) or multiple tumors, or for treating HCC involving macrovascular invasion shows the median OS investigating HCC with macrovascular invasion was approximately 50% at 1 year and 18% at 5 years 19, which were significantly higher than those in our study. And those results may benefit from that there were no extrahepatic metastasis for patients suit for HR. However, in our study, there were 19.0% of the patients accompanied with extrahepatic metastasis and 36.2% of the patients had tumor thrombosis extending to the main portal vein, the splenic vein, or the superior mesenteric vein (grades III/IV PVTT).

Drug-eluting bead TACE (DEB-TACE), a novel TACE that uses microspheres as both drug carriers and embolization agents, possesses several advantages over cTACE, including more constant drug release and better embolization effect 21-23. Accumulating evidence reveals that DEB-TACE is superior to cTACE in improving treatment response and OS but fewer adverse events of unresectable HCC patients 21, 23-25. As to its efficacy in treating HCC patients with PVTT, only two studies have been reported 26-27. In a recent study, 4% of HCC patients with PVTT achieve CR after DEB-TACE treatment, and the ORR is 26%; besides, median survival is 10 months 26. In another study, the survival of HCC patients with PVTT is also evaluated after DEB-TACE treatment, which reveals that the median OS is 3.33 months 27. Nevertheless, only two small-sample studies are unable to fully illustrate the efficacy of DEB-TACE in treating HCC patients with PVTT. Therefore, additional investigations are needed to reveal the clinical response and survival of DEB-TACE in treating HCC patients with PVTT.

CSM, the first DEB-TACE product in China, not only has general advantages of DEB-TACE, but also has higher drug loading efficiency compared with other DEB-TACE products 28. Since CSM is approved in 2015, it has been widely used in China to treat unresectable HCC patients due to its good characteristics 7, 28. Considering that the efficacy of DEB-TACE in treating HCC patients with PVTT remains to be illustrated, especially for DEB-TACE using CSM, we conducted the current study, and found that 5.2% of tumor achieved CR, and the ORR was 79.3% for tumor after DEB-TACE treatment; besides, the median PFS and OS of patients were 5.0 months and 9.0 months. The 3-, 6-, 9-, 12-, 18- and 24-month OS rates were 94.8%, 72.4%, 53.4%, 41.4%, 22.4% and 19.0%, respectively. These findings were similar to our previous study with portal vein stenting combined with iodine-125 seed strand endovascular implantation followed by TACE for treating HCC patients with PVTT 29. The efficacy of DEB-TACE in our study was better than that of the two previous studies, which might be explained by that: (1) the better properties of CSM over other DEB-TACE products contributed to the better efficacy; (2) our study only recruited patients with Child-Pugh classification A or B liver function, while previous studies also recruit Child-Pugh classification C patients, implying that HCC patients with PVTT in our study had better baseline liver function compared to the previous studies, which might also lead to the better clinical outcomes; (3) there were only 34 cases underwent DEB-TACE monotherapy, and the remaining 24 patients accepted combination therapy including sorafenib, surgery and even liver transplantation, which may prolong OS in our cohort Taken together, our study revealed that DEB-TACE using CSM presented with a favorable clinical efficacy in HCC patients with PVTT.

A lot of risk factors for OS of HCC patients with PVTT have been discovered 29. A prospective study investigates the prognostic factors of HCC patients with PVTT who receive TACE or conservative treatment, which shows that the presence of main portal vein obstruction, tumor number ≥2, TBIL ≥20 μmol/L and tumor diameter ≥11.1 cm are associated with worse OS 16. Another retrospective study including HCC patients with PVTT treated with DEB-TACE or radioembolization (90Y) revealed increased age, the extent of liver involvement and weight to be independent risk factors for poorer OS 26. More interestingly, a retrospective study discloses that Child-Pugh classification C and tumor burden >50% are independent predictors for poorer OS in HCC patients with PVTT who receive DEB-TACE or cTACE treatment 27. Consistent with previous studies, our study found that the higher Child-Pugh classification, extrahepatic metastasis and the greater tumor size as well as the higher tumor burden were associated with worse OS; what's more, the higher Child-Pugh classification and tumor burden were independent predictors for poorer OS. The possible reasons might be as follows: (i) Compared to patients at Child-Pugh classification A, patients at stage B have more dysregulated liver functions and deteriorated quality of life, thus they tend to have poorer OS; (ii) increased tumor burden indicates larger tumor size while less hepatic functional reserve, as a result, patients with increased tumor burden are also more likely to have a poorer OS. Above all, both worse liver function of Child-Pugh classification and higher tumor burden were independent risk factors for worse OS.

As for AEs of DEB-TACE in treating HCC patients with PVTT, two studies have been reported 26-27. In one of the studies, the AEs included pain, nausea and vomiting, which occurred in 10.8% of patients, and 26% of patients were dead three months post treatment 26. In the other study, post-embolization syndrome, encephalopathy, diarrhea and abnormal liver functions were the main AEs, and 6.3% of patients were dead one month post treatment 27. In our study, the most common clinical AEs were PES and the most prevalent laboratory toxicity was transient liver function damage. There were no deaths occurred within 1 month after treatment but three patients died due to hepatic failure or gastrointestinal bleeding within 3 months after the procedure. There could be many reasons for the lower mortality rate compared with previous studies but the main two reasons were the patients in our cohort with Child-Pugh classification A or B liver function and successful superselective catheterization of not only tumor vessels but also the nutrient arteries contributing to tumor thrombosis. Our results indicated that DEB-TACE with CSM had good safety for HCC with PVTT in patients with Child-Pugh classification A or B liver function.

There were some limitations in the current study. Firstly, the study was a single-arm study without comparison, and the sample size was relatively small, thus the randomized, control study with a larger sample size is needed. Second, all of the patients recruited in this study were treatment-naïve patients, so the efficacy and safety of DEB-TACE using CSM in patients with treatment-experienced remained unclear. Third, only a few patients in our study combined with sorafenib, and the most common reason is the high cost that patients cannot afford because sorafenib is not on the list of drugs covered by medical insurance of China until October 2017. Therefore, the treatment outcome of DEB-TACE using CSM combined with sorafenib in the real-world needs further study. At last, we only evaluated the efficacy and safety of DEB-TACE using CSM in Chinese patients, which could not represent other race patients, thus the efficacy and safety of DEB-TACE using CSM in other race patients need further investigated in the future.

In summary, DEB-TACE is safe and well-tolerated in HCC patients with PVTT, and reveals a good preliminary clinical outcome according to our study, although requiring further stringent evaluation. The higher Child-Pugh classification and liver tumor burden are independent prognostic factors associated with poor survival for HCC patients with PVTT treated by DEB-TACE with CMS.

## Figures and Tables

**Table 1:** Patients' baseline characteristics (N=58).

Characteristics	No.	Percentage	Characteristics	No.	Percentage
Age (years)			2	2	3.4
≥60	23	39.7	≥3	39	67.3
<60	35	60.3	Tumor type		
Gender			Nodular	11	19
Male	53	91.4	Massive	11	19
Female	5	8.6	Diffuse	36	62
ECOG performance status			Maximum tumor size (cm)		
0	22	37.9	<5	7	12.1
1	36	62.1	≥5, <10	28	48.3
Child-Pugh classification			≥10	23	39.6
A	47	81	Tumor burden		
B	11	19	≤30%	18	31
Viral etiology			≤50%	20	34.5
HBsAg (+)	52	90	>50%	20	34.5
HBsAg (-)	6	10	Serum AFP (ng/ml)		
HBV-DNA(IU/ml)			<200	22	37.9
Below the detection limit	26	44.8	≥200, <400	3	5.2
<10^4^	13	22.4	≥400	33	56.9
≥10^4^	19	32.8	Type of PVTT		
Liver cirrhosis			I	16	27.6
Yes	56	96.6	II	21	36.2
No	2	3.4	III	16	27.6
Ascites			IV	5	8.6
Yes	11	19	Extrahepatic metastasis		
No	47	81	Yes	11	19
No. of HCC nodules			No	47	81
1	17	29.3			

Data were presented as count and percentage. ECOG, Eastern Cooperative Oncology Group; HBsAg, Hepatitis B surface antigen; HBV; Hepatitis B Virus; HCC, hepatocellular carcinoma; AFP, alpha-fetoprotein; PVTT, Portal Vein Tumor Thrombus.

**Table 2:** Response to treatment.

Items	CR	PR	SD	PD	ORR
Tumor	3 (5.2)	43 (74.1)	9 (15.6)	3 (5.1)	46 (79.3)
Tumor Thrombus	2 (3.4)	24 (41.4)	25 (43.1)	7 (12.1)	26 (44.8)

Date were presented as count (percentage). CR, complete remission; PR, partial remission; SD, stable disease; PD, progression disease; ORR, overall response rate (ORR=CR+PR).

**Table 3:** Univariate and stepwise multivariate Cox's proportional hazards regression model analysis factors affecting OS.

Variable	Univariate analysis			Multivariate analysis		
	HR	95% CI	*P*	HR	95% CI	*P*
Age	0.970	0.485-1.940	0.932	-	-	-
Gender (male/female)	1.387	0.331-5.807	0.654	-	-	-
Etiology(hepatitis infection/other)	0.760	0.267-2.167	0.608	-	-	-
HBV-DNA detectable(yes/no)	1.170	0.805-1.700	0.412	-	-	-
Child-Pugh classification (B/A)	2.986	1.412-6.316	0.004	2.279	1.042-4.985	0.039
ECOG (0/1)	0.713	0.347-1.464	0.357	-	-	-
Extrahepatic metastasis (yes/no)	2.481	1.142-5.393	0.022	-	-	-
Maximum tumor size			0.009			
<5	Ref					
≥5, <10	1.986	0.447-8.819	0.367			
≥10	5.081	1.173-22.002	0.030			
No. of HCC nodules (≥3/<3)	1.217	0.818-1.811	0.333	-	-	-
AFP (≥400/<400)	1.168	0.810-1.683	0.407	-	-	-
Type of PVTT			0.813	-	-	-
I	Ref					
II	1.148	0.469-2.813	0.762			
III	1.539	0.615-3.850	0.357			
IV	1.135	0.3000-4.285	0.852			
Tumor type			0.141	-	-	-
Nodular	Ref					
Massive	2.065	0.729-5.851	0.172			
infiltrative	0.929	0.370-2.328	0.874			
Tumor Burden			0.002			0.008
≤30%	Ref			Ref		
≤50%	3.273	1.147-9.337	0.027	3.300	1.152-9.454	0.026
>50%	6.248	2.228-17.523	0.000	5.380	1.873-15.458	0.002
Ascites (yes/no)	1.898	0.850-4.235	0.118	-	-	-

Data were presented as P value, HR (hazards ratio) and 95% CI (confidence interval). Factors affecting OS (overall survival) were determined by univariate and multivariate Cox's proportional hazards regression analyses. *P* value <0.05 was considered significant. “-” indicated that the factor was no statistical significance. ECOG, Eastern Cooperative Oncology Group; HBsAg, Hepatitis B surface antigen HBV; Hepatitis B Virus; HCC, hepatocellular carcinoma; AFP, alpha-fetoprotein; PVTT, Portal Vein Tumor Thrombus.

**Table 4:** The main AEs of DEB-TACE with CSM for HCC with PVTT.

Variables	Grade 1-2 (n, %)	Grade 3-4 (n, %)
Abdominal pain	44 (75.9)	1 (1.7)
Fever	53 (91.4)	2 (3.4)
Nausea	41 (70.7)	0
Diarrhea	5 (8.6)	0
WBC decreased	6 (10.3)	0
Neutrophils decreased	2 (3.4)	0
HGB decreased	3 (5.2)	0
PLT decreased	15 (25.9)	6 (10.3)
Elevated ALT	29 (50.0)	21 (36.2)
Elevated AST	22 (37.9)	34 (58.6)
Hyperbilirubinemia	37 (63.8)	2 (3.4)

Note: WBC, white blood cell; HBG, hemoglobin; PLT, platelet; ALT, alanine aminotransferase; AST, aspartate aminotransferase; GI, gastrointestinal.

## Abbreviations

DEB: Drug-eluting beads
TACE: Transcatheter arterial chemoembolization
CSM: CalliSpheres^®^ microsphere
HCC: Hepatocellular carcinoma
PVTT: Portal vein tumor thrombus
AASLD: the American Association for the Study of Liver Diseases
mRECIST: modified Response Evaluation Criteria in Solid Tumors
AEs: Adverse events
OS: Overall survival
CR: Complete response
ECOG: Eastern Cooperative Oncology Group
ORR: Overall response rate
PFS: Progression free survival
PR: Partial response
SD: Stable disease
PD: Progressive disease
CT: Computed Tomography
MRI: Magnetic Resonance Imaging
PVA: Polyvinyl alcohol
